# Atrial Cardiomyopathy in Patients with Cardiovascular-Kidney-Metabolic Syndrome: Non-Invasive Characterization (ATRIO-CKM)

**DOI:** 10.3390/diagnostics16193196

**Published:** 2026-10-01

**Authors:** Maria-Mihaela Godun, Marius-Traian-Dragoș Marcu, Paula Cristina Morariu, Alexandru-Florinel Oancea, Mugurel-Costel Apetrii, Anca-Elena Ștefan, Diana-Elena Floria, Laura Huiban, Bogdan Todiraș, Mariana Pavel-Tănasă, Anca Trifan, Adrian Covic, Mariana Floria

**Affiliations:** 1 Grigore T. Popa University of Medicine and Pharmacy Iași, 700115 Iași, Romania; 2 Internal Medicine Clinic, “Sf. Spiridon” County Clinical Emergency Hospital, 700111 Iași, Romania; 3 Clinical Hospital of Pneumology, 700115 Iași, Romania; 4 Nephrology Clinic, “Dr. C.I. Parhon” Clinical Hospital, 700503 Iași, Romania; 5 Institute of Gastroenterology and Hepatology, “Sf. Spiridon” County Clinical Emergency Hospital, 700111 Iași, Romania; 6 Laboratory of Immunology, Department of Immunology, “Sf. Spiridon” County Clinical Emergency Hospital, 700111 Iași, Romania

**Keywords:** atrial cardiomyopathy, cardiovascular-kidney-metabolic syndrome, left atrial strain, interatrial block, MR-proANP, Fetuin-A, speckle-tracking echocardiography, cardiac magnetic resonance

## Abstract

**Background/Objectives**: In cardiovascular-kidney-metabolic (CKM) syndrome, left atrium (LA) involvement begins early: reservoir function declines before the chamber dilates, and the resulting atrial cardiomyopathy (ACM) precedes atrial fibrillation (AF), heart failure with preserved ejection fraction (HFpEF), and cardioembolic stroke. How ACM is distributed across CKM stages has not yet been characterized in routine clinical practice. ATRIO-CKM aims to characterize ACM non-invasively across the range of CKM stages in patients assessed in an internal medicine and cardiology department and to identify the parameters most strongly associated with ACM within CKM syndrome. **Methods**: Single-center, prospective, observational study with cross-sectional phenotyping at the index visit. We will enroll consecutive adults at their first assessment in our department (target, 200; minimum, 150). The primary objective is to estimate the stage-specific prevalence of ACM among participants in sinus rhythm without documented AF, who form the main cohort and are grouped as CKM with versus without ACM. Patients in sinus rhythm are phenotyped with 12-lead electrocardiogram (P-wave duration, Bayés interatrial block), echocardiography with speckle-tracking of the left ventricle (LV), LA (biplane reservoir, conduit, and contractile strain), and right ventricular (RV) free wall, and biomarkers including MR-proANP, Fetuin-A, FGF-23, and NT-proBNP, with cardiac magnetic resonance in an exploratory subset. Participants with AF documented at any time, including previously documented paroxysmal AF, are allocated to a separate established-AF cohort irrespective of the rhythm at the index visit. **Results**: We will report ACM prevalence by CKM stage with confidence intervals, the profile separating the two groups, LA volume index across three groups of increasing severity (no ACM, ACM in sinus rhythm, and established AF), and the parameters associated with ACM. **Conclusions**: Using only measurements already available in routine cardiology, ATRIO-CKM will describe how ACM is distributed across CKM stages and identify the parameters most strongly associated with ACM within CKM syndrome, as candidates for a classification index to be developed and validated in a larger cohort.

## 1. Introduction

Contemporary cardiology and internal medicine practice is increasingly characterized by the management of patients with multimorbidity. Clinical manifestations range from an excess of adiposity or dysglycemia, through hypertension and a slowly rising creatinine, to a heart that has already begun to remodel. The American Heart Association (AHA) gave this interconnected spectrum of disorders a name in 2023: cardiovascular-kidney-metabolic (CKM) syndrome. This moment marked a paradigm shift by unifying cardiovascular, metabolic, and kidney disorders within a single continuum of pathologies, replacing the traditional organ-specific approach [1]. Cardiovascular-kidney-metabolic syndrome represents a growing public health challenge. The prevalence of adults with at least one CKM condition increased from 21.2% in 1999 to 26.3% in 2020. In addition, data from 11,607 National Health and Nutrition Examination Survey participants showed that a quarter had at least one cardiac, renal, or metabolic disorder, whereas the prevalence of multimorbidity was 8% in the overall population and 25% among individuals aged ≥65 years [2]. In the absence of appropriate management, CKM syndrome is associated with excess mortality, increased rates of preventable hospitalization, and substantial healthcare costs, predominantly attributable to cardiovascular disease [3].

The left atrium (LA) appears to be affected early in the course of CKM syndrome. Functional impairment, particularly of the reservoir function, may develop before the atrial enlargement becomes evident, reflecting the cumulative effects of adiposity-related inflammation, insulin resistance, elevated filling pressure, and early renal dysfunction [1,2,3,4]. The early functional deterioration represents one of the earliest detectable manifestations of atrial cardiomyopathy (ACM)–the term covering any structural, architectural, contractile, or electrical abnormality of the atria associated with adverse clinical outcomes, including atrial fibrillation (AF), heart failure with preserved ejection fraction (HFpEF), and ischemic stroke [5]. Consequently, the LA may serve as an early marker of the systemic cardiovascular, renal, and metabolic dysfunction that characterizes CKM syndrome, reflecting increased hemodynamic load, myocardial stiffness, and progressive atrial fibrosis, before the enlargement becomes apparent.

The metabolic aspect of this relationship has become increasingly evident. Fetuin-A, a hepatocyte-derived glycoprotein and a marker of metabolic syndrome, is elevated in patients with AF independently of the usual risk factors [6,7], which places a circulating biomarker directly in the atrial remodeling pathway. Whether this translates into a therapeutic target for ACM specifically remains to be confirmed: sodium–glucose cotransporter 2 (SGLT2) inhibitors and glucagon-like peptide-1 (GLP-1) receptor agonists–originally glucose-lowering agents now used across the cardiac, renal, and metabolic spectrum–have demonstrated anti-inflammatory and reverse-remodeling effects important to CKM syndrome [8], but their direct benefit on ACM has yet to be established. Recent cardiac magnetic resonance (CMR) data show that atrial fibrosis is already present in patients at high predicted risk of AF, before the arrhythmia becomes clinically manifested [9], supporting the idea that atrial substrate can be identified before the disease becomes overt.

Mid-regional pro-atrial natriuretic peptide (MR-proANP) has attracted increasing interest as a biomarker of atrial remodeling and dysfunction. In contrast to B-type natriuretic peptide and N-terminal pro-B-type natriuretic peptide (NT-proBNP), which reflect myocardial stress in both the atria and the ventricles, MR-proANP is more closely associated with LA remodeling, atrial volume overload, and mechanical dysfunction. Accordingly, MR-proANP may complement the early characterization of ACM [10,11].

What is missing is the step of putting these pieces together. Metabolic syndrome is well established as a risk factor for AF and stroke, yet how CKM syndrome and ACM contribute jointly has barely been examined [12]. Data from implanted devices show that episodes of subclinical AF lasting more than six minutes are rarely captured in the month before a stroke or systemic embolism occurs [13]. In practice, this means that by the time an arrhythmia becomes apparent, most of the window during which the underlying atrial substrate could have been identified has already passed. Nevertheless, ACM can be recognized earlier than that, at the point a patient reaches a specialist assessment, and, importantly, with instruments already available in most cardiology clinics. This would allow the underlying process to be recognized and addressed, rather than waiting for stroke, AF, or heart failure to occur.

The ATRIO-CKM study is built around this idea. We propose to characterize the atrial substrate non-invasively, at the index visit, using three complementary assessments: the 12-lead electrocardiogram (P-wave morphology and duration, Bayés interatrial block), advanced transthoracic echocardiography with automated speckle-tracking of LA deformation, and a targeted biomarker panel. Aligned with CKM stages, these measures should show in which CKM stages ACM is present and identify the parameters most strongly associated with ACM within CKM syndrome, as candidates for a classification index to be developed and validated in a larger cohort. This article details the protocol of the ATRIO-CKM study.

## 2. Experimental Design

### 2.1. Study Design and Rationale

ATRIO-CKM is a single-center, prospective, observational study in which phenotyping is done once, at the index visit. Patients are enrolled at the first assessment in our department. The patients can already have established metabolic, renal, or cardiovascular conditions, and they enter the study spread across the CKM stages, in practice from stage 1 upwards, since stage 0 by definition describes people without the risk factors that bring patients to an internal medicine and cardiology specialist. All measurements are taken at this single visit, except CMR, which is scheduled as scanner capacity permits (schedule of assessments in Table S1). At entry, we record CKM stages, cardiovascular risk factors, biomarkers, and the ACM phenotype. We chose a cross-sectional design because the primary question can be answered without follow-up: to see how ACM is distributed across the CKM stages in patients referred for specialist assessment, one standardized evaluation is enough.

To ensure transparency and allow the planned design and outcomes to be verified against the final report, the study was prospectively registered at ClinicalTrials.gov (identifier NCT07697469) on 5 July 2026, before any participant was enrolled. Recruitment started on 10 July 2026, and its estimated completion date is October 2027. Reporting of this protocol has been mapped to the Strengthening the Reporting of Observational Studies in Epidemiology (STROBE) statement (Table S2).

Cohort allocation is based on documented AF rather than on the rhythm recorded at the index visit. Any participant with AF documented at any time before or at the index visit (definition in Section 2.4), including previously documented paroxysmal AF, is allocated to the established-AF cohort; the primary cohort comprises participants in sinus rhythm at the index visit who have no documented history of AF, and these participants are further categorized based on direct measurements of ACM status. Within this cohort, the main comparison is between CKM participants with and without ACM. In a patient in sinus rhythm at the index visit, we obtain the full phenotype: P-wave duration, interatrial block, and the reservoir, conduit, and contractile components of atrial strain, and, in participants in the sinus-rhythm cohort, we apply the criteria to confirm or exclude ACM. In a patient who is in AF at the index visit, these measurements cannot be made, but AF represents a clinical expression of ACM and is also part of CKM stage 4. Participants allocated to the established-AF cohort who are in sinus rhythm at the index visit undergo the same full assessment as the primary cohort; their P-wave and atrial strain measurements are recorded and reported descriptively as a prespecified subgroup (Section 2.7), but they are not included in the primary prevalence denominator, because previously documented AF already establishes the clinical expression of ACM that the primary analysis is intended to detect before it becomes manifest.

Participants of the established-AF cohort are therefore presented separately and excluded from the primary prevalence and association analyses. As a secondary analysis, a comparison is performed across three levels of increasing severity: no ACM, ACM in sinus rhythm, and established AF.

### 2.2. Objectives

The primary objective is to estimate the prevalence of ACM within each CKM stage represented in the study population, using non-invasive parameters, in patients assessed at their index visit to a specialist internal medicine and cardiology service. Stages 1 to 4 are expected in this setting, for the reason given in Section 2.1; stage 0 will be reported only if participants in that stage are recruited.

The secondary objectives are to (i) describe the clinical, laboratory, electrocardiographic, echocardiographic, and therapeutic profile of ACM within CKM syndrome; (ii) compare LA function across CKM stages; (iii) examine the relationship between Bayés interatrial block on the electrocardiogram (ECG) and CKM syndrome, and explore LA remodeling according to SGLT2-inhibitor or GLP-1 receptor-agonist use as an exploratory cross-sectional association; (iv) identify the parameters associated with ACM within CKM syndrome.

### 2.3. Population and Eligibility

Adults presenting for a first cardiovascular evaluation in our department (internal medicine and cardiology).

Inclusion: age ≥18 years; presentation for cardiovascular evaluation; signed written informed consent and agreement with protocol requirements.

Exclusion: data insufficient to assign a CKM stage; valvular disease greater than moderate; mechanical or biological valve prosthesis; temporary or permanent cardiac pacing; known cardiomyopathy (hypertrophic, dilated, restrictive, or infiltrative); active or untreated thyroid disease; significant psychiatric disease; refusal or inability to comply.

Eligible participants in sinus rhythm at the index visit and without documented AF are assigned to CKM with ACM (study group) or CKM without ACM (control group); participants with AF documented at any time, including previously documented paroxysmal AF, form the separate established-AF cohort (Section 2.4).

### 2.4. A Priori Definition of ACM and CKM Staging

**Figure 1:**
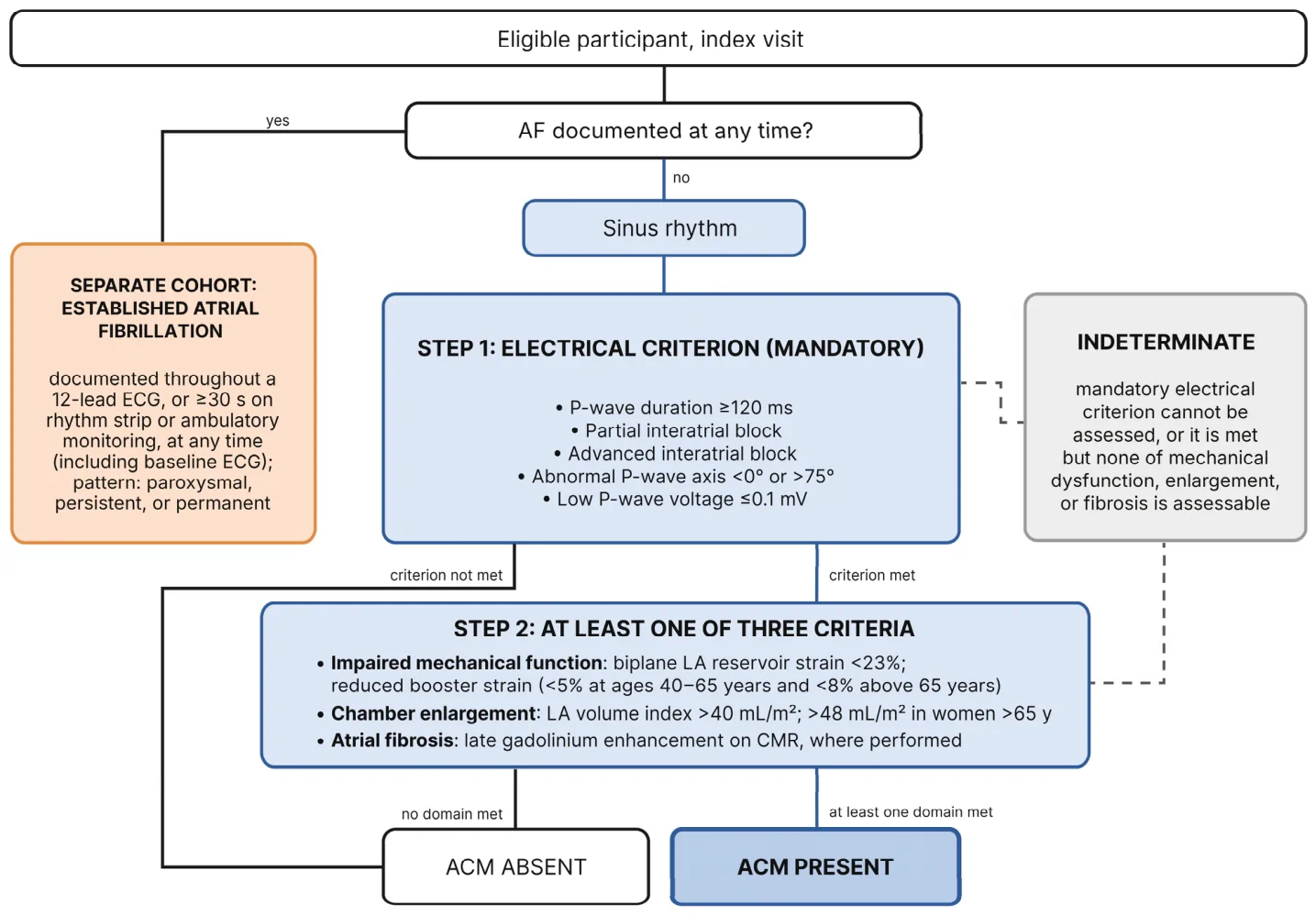
Algorithm for the classification of atrial cardiomyopathy (ACM), adapted from the 2025 clinical consensus statement of the Heart Failure Association of the ESC [16]. Among participants in the sinus-rhythm cohort, that is, those in sinus rhythm at the index visit with no documented AF, those who meet the Step 1 mandatory electrical criterion (P-wave abnormalities) are further investigated with echocardiography or CMR. If impaired mechanical function, chamber enlargement, or atrial fibrosis is identified, the participant is classified as having ACM. Participants in whom the Step 1 electrical criterion cannot be assessed, or in whom it is met but Step 2 is not assessable, are classified as indeterminate. Participants who meet the definition of established AF are not classified by this algorithm and form a separate cohort; allocation to that cohort is based on documented AF (Section 2.4), not on the rhythm recorded at the index visit. ACM, atrial cardiomyopathy; AF, atrial fibrillation; ECG, electrocardiogram; LA, left atrium; CMR, cardiac magnetic resonance.

At present, there is no universally accepted diagnostic standard for ACM. Several groups have proposed criteria that overlap without matching: the EHRA/HRS/APHRS/SOLAECE consensus and its 2024 revision [5,14], the ARCADIA trial definition [15], and the 2025 statement from the Heart Failure Association (HFA) of the European Society of Cardiology (ESC) [16]—each calling its own thresholds provisional. In our study, we will use the most recent HFA/ESC 2025 framework, and treat it as a proposal rather than a settled standard. Under this framework, this a priori definition is operationalized through the classification algorithm in Figure 1, which assigns each participant of the sinus-rhythm cohort to ACM-present, ACM-absent, or indeterminate status; participants who meet the definition of established AF are not classified by this algorithm, since AF itself represents a clinical expression of ACM and is analyzed as a separate cohort (Section 2.1). The diagnosis requires the electrical criterion to be met, plus at least one sign of impaired mechanical function, chamber enlargement, or atrial fibrosis. The reason for requiring both is practical: a P wave can be prolonged, or an atrium dilated, for reasons unrelated to ACM, and combining an electrical with a structural abnormality keeps the false-positive rate down.

Step 1—electrical dysfunction (required): the P wave is assessed with the consensus ACM P-wave criteria. The criterion is met by any of the following: a P-wave duration ≥120 ms; partial interatrial block (≥120 ms, bimodal in the inferior leads); advanced interatrial block (≥120 ms with a biphasic pattern in two or more inferior leads); an abnormal P-wave axis (<0° or >75°); low P-wave voltage (≤0.1 mV). Higher grades reflect more advanced disease—advanced interatrial block alone, then advanced block with paroxysmal arrhythmia, then with persistent arrhythmia—so the grading also serves as a measure of severity [16,17].

Step 2—at least one imaging criterion required. Mechanical LA dysfunction: atrial function is considered impaired when biplane LA reservoir strain (LASr) falls below 23%; a reduced contractile (booster) strain (absolute value <5% at ages 40–65 years and <8% above 65 years) supports this in sinus rhythm. Both thresholds are those of the HFA/ESC statement [16]; they were derived from 2D speckle-tracking with ventricular end-diastole as the reference, which is the standard technique in the Strain+ package used in this study, so vendor-specific offsets are stated as a limitation. Enlargement is defined from the two-dimensional LA volume index (LAVI). We follow the consensus numbers: LAVI above 40 mL/m^2^ marks enlargement, and the threshold is 48 mL/m^2^ in women over 65 years [16]. When CMR and the fibrosis quantification software are available, atrial fibrosis on late gadolinium enhancement (LGE; Section 4.7) serves the same purpose.

To keep the cohort complete across definitions, we also record the parameters specific to the other schemes: LA emptying fraction and NT-proBNP for the four-stage staging system [18,19].

These criteria apply to participants in sinus rhythm at the index visit who have no documented history of AF. Established AF is defined as AF documented throughout a standard 12-lead ECG, or for at least 30 s on a single-lead tracing (rhythm strip or ambulatory monitoring), at any time before or at the index visit, including the baseline ECG; the pattern is recorded as paroxysmal, persistent, or permanent according to current definitions. Participants who meet this definition are not classified by the algorithm above and form the established-AF cohort irrespective of the rhythm recorded at the index visit, since a previously documented episode already identifies the atrial disease that the algorithm is intended to detect in its preclinical stage.

CKM stage (0–4) is then assigned according to the AHA framework and current staging descriptions [1,3], which integrate metabolic risk factors, clinical kidney disease, and cardiovascular disease. The primary comparison remains between CKM patients with and without ACM.

If the Step 1 electrical criterion cannot be assessed, or if it is met but none of the Step 2 criteria—mechanical dysfunction, chamber enlargement, or atrial fibrosis—is assessable, the participant is classified as indeterminate. Indeterminate participants are counted separately and shown in the participant flow diagram in the final study report (Figure S1 shows the planned flow structure).

### 2.5. Outcome Measures

Because assessment is cross-sectional, all outcomes are defined at baseline.

Primary: prevalence of ACM in the sinus-rhythm cohort, reported separately for each CKM stage represented in the study population, as a percentage of the stage-specific denominator with 95% Wilson confidence intervals. Stages are not combined: a stage with a small number of participants is reported with its own denominator and confidence interval and identified as imprecise, rather than merged with an adjacent stage.

Secondary: biplane LA reservoir strain across CKM stages; prevalence and grade of Bayés interatrial block by stage; serum concentration of MR-proANP, Fetuin-A, fibroblast growth factor 23 (FGF-23), and NT-proBNP across ACM and CKM categories; and the parameters associated with ACM within CKM syndrome, reported as exploratory.

### 2.6. Sample Size

We aim to enroll approximately 200 participants, no fewer than 150, limited by the attendance of patients at the department over the recruitment period. For a single proportion, the half-width of the 95% confidence interval is maximal when the observed proportion is 50%: 200 participants estimate the prevalence of ACM to within ±6.9 percentage points and 150 to within ±7.9 percentage points. This is adequate for the primary objective, which is to describe how ACM is distributed across CKM stages. Precision for the primary outcome depends on the number of participants within each CKM stage rather than on the overall sample. Stages with small numbers will be reported with their confidence intervals and identified as imprecise. For example, a stage represented by 50 participants would be estimated to within ±13.4 percentage points.

The number of covariates that can be reliably included in a model is determined by the number of participants meeting the ACM definition, rather than by the total sample size. The prevalence of ACM has been reported under other definitions and in other populations, but no estimate exists under the definition applied here or by CKM stage; establishing it is an objective of this study. At the index visit, demographic data and the parameters specified in Section 4 will be collected; other covariates previously proposed (blood pressure, adiposity, glycemia, estimated glomerular filtration rate (eGFR) and lipid profile) are themselves components of the CKM staging definition, so adjusting for them would condition on the exposure.

### 2.7. Statistical Analysis

Statistical analyses will be performed in IBM SPSS Statistics version 31.0 (IBM Corp., Armonk, NY, USA), with two-sided tests at α = 0.05 and estimates reported with 95% confidence intervals. The following procedures will be used: One-Sample Proportions (Wilson score intervals); LOGISTIC REGRESSION (binary logistic regression, including the Hosmer–Lemeshow test, Cook’s distance, and leverage values); PLUM (ordinal regression, including the test of parallel lines); REGRESSION (linear regression and collinearity diagnostics) and QUANTREG (quantile regression); MULTIPLE IMPUTATION (IBM SPSS Missing Values module); and RELIABILITY (intraclass correlation coefficients). Restricted cubic spline terms and Bland–Altman limits of agreement will be computed with SPSS syntax. Continuous variables will be summarized as mean ± standard deviation or median with interquartile range according to distribution, and categorical variables as counts and percentages; distributional assumptions will be checked, and non-parametric methods used where appropriate. Missing data will be handled as set out in Section 2.8 (see below).

The primary analysis will model the prevalence of ACM within each CKM stage represented, among participants in the sinus-rhythm cohort, with stage-specific denominators and 95% confidence intervals. In the secondary adjusted analysis, we will use logistic regression to test whether ACM is more frequent at more advanced CKM stages. CKM stage is the explanatory variable, and each stage is compared with the lowest stage represented, with age and sex as prespecified covariates. The other candidate covariates (blood pressure, body mass index (BMI) or waist circumference, glycemia or glycated hemoglobin (HbA1c), eGFR, and lipid profile) are components of the CKM staging definition and are therefore not entered into this model (Section 2.6). Results will be reported as odds ratios with 95% confidence intervals. Secondary analyses will include linear or quantile regression of left atrial reservoir strain on CKM stage. Within the biomarker family (MR-proANP, Fetuin-A, FGF-23, NT-proBNP), several biomarkers are influenced by renal function; their associations with ACM will therefore be adjusted for eGFR, and, because eGFR is also a CKM staging component, these adjusted estimates will be interpreted with this overlap in mind.

A further prespecified analysis will examine left atrial size, expressed as LAVI, across three ordered levels of severity (no ACM, ACM in sinus rhythm, and established AF), using ordinal regression with severity level as the outcome and LAVI as the explanatory variable.

Model assumptions and diagnostics are prespecified as follows. Nonlinearity: for continuous covariates (age in the logistic model, eGFR in the biomarker models, and LAVI in the ordinal model), the linear term will be compared with a restricted cubic spline with three knots at the 10th, 50th, and 90th percentiles using a likelihood ratio test; the spline term will be retained if *p* < 0.05, and the linear term otherwise. Collinearity: variance inflation factors (VIF) will be obtained from collinearity diagnostics, and a VIF above 5 will be taken to indicate relevant collinearity, in which case the correlated covariates will not be entered simultaneously. Calibration: calibration of the logistic model will be assessed with a calibration plot of observed versus predicted probabilities across deciles of predicted probability and with the Hosmer–Lemeshow test; *p* < 0.05 will be taken to indicate inadequate fit, which will be reported, and the model specification will be re-examined. Ordinal model: the proportional odds assumption will be assessed with the test of parallel lines; if it is violated (*p* < 0.05), a multinomial logistic model will be fitted as a sensitivity analysis. Linear model: normality and homoscedasticity of residuals will be inspected graphically, and median (quantile) regression will be used if these assumptions are not met. Influential observations: observations with Cook’s distance > 4/*n* or leverage > 2(*k* + 1)/*n*, where *n* is the number of observations and *k* the number of predictors, will be flagged; models will be refit without them as a sensitivity analysis, and both results will be reported if their exclusion changes any odds ratio by more than 10% or alters its statistical significance.

Finally, associations between the recorded parameters and ACM within CKM stages will be reported as hypothesis-generating, with the number of comparisons stated. No adjustment for multiplicity is planned for these exploratory analyses, including the biomarker analyses; nominal *p*-values and confidence intervals will be reported together with the number of comparisons performed, and no inference about individual parameters will be drawn from them. The parameters most strongly associated with ACM will be reported as candidates from which a classification index could be formed and validated in the future, in a cohort large enough to support it. No diagnostic score is derived, fitted, or internally validated in the present cohort: the sample size, the uncertain number of ACM events, and the fact that several candidate electrocardiographic and echocardiographic variables are themselves components of the ACM definition would make such a model both unstable and partly circular. Participants in the established-AF cohort who are in sinus rhythm at the index visit are described separately (P-wave indices, the three LA strain components, LAVI, and biomarkers), without being included in the primary prevalence analysis.

### 2.8. Missing Data

Three situations are distinguished: variables whose absence prevents enrollment, measurements that are structurally absent, and ordinary missing data. Missingness of the variables required to assign a CKM stage prevents classification and is an exclusion criterion at screening (Section 2.3); these participants do not enter the study and are not imputed. If a variable required for staging nevertheless cannot be obtained after enrollment, the participant is excluded from the analyses and reported in the participant flow diagram, without imputation. Unclassifiable participants are excluded from the prevalence denominator and reported in the participant flow diagram: ACM status is indeterminate where the Step 1 mandatory electrical criterion cannot be assessed, or where it is met but Step 2 imaging (echocardiography or CMR) cannot be assessed. The primary analysis is therefore a complete-case analysis by definition. Two prespecified sensitivity analyses bound the effect of this exclusion: in the first, all indeterminate participants are counted as ACM-absent, and in the second, all are counted as ACM-present; both bounds are reported for each CKM stage alongside the primary estimate.

Structurally absent measurements are not missing data and are not imputed. In participants who are in AF at the index visit, the P-wave indices and the conduit and contractile strain components cannot be measured; these values are structurally absent rather than missing, and such participants form part of the established-AF cohort (Section 2.4). CMR is performed in an exploratory subset only; CMR variables are not imputed, and CMR-based analyses are restricted to participants who underwent the examination.

Variables eligible for imputation are those collected after enrollment that may be missing for technical or administrative reasons in a participant who remains classifiable for ACM status: anthropometric and blood-pressure measurements, laboratory values other than those required for CKM staging, individual biomarker concentrations, individual P-wave indices, and individual echocardiographic parameters. In the adjusted analyses, where such data are missing, we will use multiple imputation by chained equations with 20 imputations, combined by Rubin’s rules and restricted to the sinus-rhythm cohort. The model will include ACM status, CKM stage, age, sex, and the variables of the analysis model. Imputation assumes missingness at random, which we cannot verify, so a complete-case analysis will be performed as a sensitivity analysis, and imputed and complete-case results will be reported side by side.

## 3. Materials and Equipment

- Anthropometry and vital signs: calibrated scale (BMI), validated oscillometric blood pressure measurement device.
- Electrocardiography: CARDIO M-PLUS 12-lead electrocardiograph (medical ECONET GmbH, Oberhausen, Germany).
- Echocardiography: Samsung cV8 cardiovascular ultrasound system (Samsung Medison, Seoul, Republic of Korea) with a phased-array transthoracic transducer. Deformation imaging uses the on-board Strain+ package, which covers all three chambers of interest: left ventricular segmental and global longitudinal strain with 17-segment bull’s-eye; LA strain reported as reservoir (LASr), conduit (LAScd), and contractile (LASct) components from apical four- and two-chamber views with biplane value; and right ventricular free-wall strain from three free-wall segments. Ejection fraction is obtained with AutoEF, the system’s deep-learning automated method, and cross-checked against the biplane Simpson measurements.
- Cardiac magnetic resonance: CMR adds tissue-level characterization of the atrial substrate (Section 4.7), but access to the scanner and to the dedicated analysis software is limited in our setting. We will therefore seek it in as many participants as scheduling permits; this component is exploratory, no formal target number is set (approximately 30 participants are expected given scanner capacity; Section 4.7), and no planned analysis depends on it.

## 4. Detailed Procedure

### 4.1. Screening and Consent

Patients attending the department are screened against the eligibility criteria at the index visit, and a screening log is kept. Written informed consent is obtained before any study-specific procedure. Cohort allocation follows the definition of established AF (Section 2.4), whereas the rhythm at the index visit determines only the extent of phenotyping, and neither affects eligibility: participants in sinus rhythm at the index visit undergo the full assessment, whether or not AF has been documented previously, whereas participants who are in AF at the index visit undergo a reduced assessment in which P-wave indices and the conduit and contractile strain components cannot be measured.

### 4.2. Clinical and Drug History

Recorded variables include age, sex, income level, smoking status, alcohol use, medical history, and the physical examination (BMI, abdominal perimeter, blood pressure, heart rate), together with a structured cardiovascular risk assessment. Drug history covers antiplatelet agents, antiarrhythmics, angiotensin-converting enzyme (ACE) inhibitors, angiotensin II receptor blockers (ARBs), as well as SGLT2 inhibitors and GLP-1 receptor agonists, for which current use at the index visit is documented as present or absent.

### 4.3. Electrocardiography

A resting 12-lead ECG is recorded at 25 mm/s and 10 mm/mV. P-wave duration is measured in the lead with the widest visible P wave, and Bayés interatrial block is graded as partial or advanced [17]. Measurement of P-wave axis, amplitude, terminal force in lead V1, and dispersion is done on the same ECG recording.

### 4.4. Blood Sampling

Venous blood is drawn at the index visit for a routine laboratory panel and for measurement of the study biomarkers, including MR-proANP, Fetuin-A, and FGF-23. Samples are centrifuged, aliquoted, and stored at −80 °C. Fetuin-A and FGF-23 are measured by enzyme-linked immunosorbent assay (ELISA) using the Human Fetuin-A ELISA (catalog number RD191037100) and the Human FGF-23 ELISA (catalog number RBL007R), respectively, both supplied by BioVendor–Laboratorni medicina a.s. (Brno, Czech Republic). MR-proANP is measured by ELISA (catalog number EH4063; Wuhan Fine Biotech Co., Ltd., Wuhan, China; supplied by Filara Biomed, Cluj-Napoca, Romania). NT-proBNP is measured in plasma by chemiluminescent enzyme immunoassay on the PATHFAST immunoanalyzer (PHC Corporation, Tokyo, Japan).

### 4.5. Transthoracic Echocardiography

Each patient undergoes a comprehensive transthoracic study, acquired and reported according to current recommendations for chamber quantification and diastolic function [20,21]. This includes left and right ventricular and bi-atrial size and function, diastolic assessment, and valve morphology.

### 4.6. Speckle-Tracking Deformation Imaging

Myocardial deformation is assessed by two-dimensional speckle-tracking on the Samsung cV8 (Strain+ package), which automatically tracks the myocardial wall and generates strain-time curves for the left ventricle, the LA, and the right ventricular free wall; each tracing is reviewed by the operator before acceptance.

Deformation loops are acquired at a frame rate of 50–100 frames per second, during three consecutive cycles, and ectopic and post-ectopic beats are excluded. A loop is accepted only if the endocardial border is visualized throughout the cycle. Participants with below-standard image quality will be classified according to the remaining ACM classification criteria.

**Figure 2:**
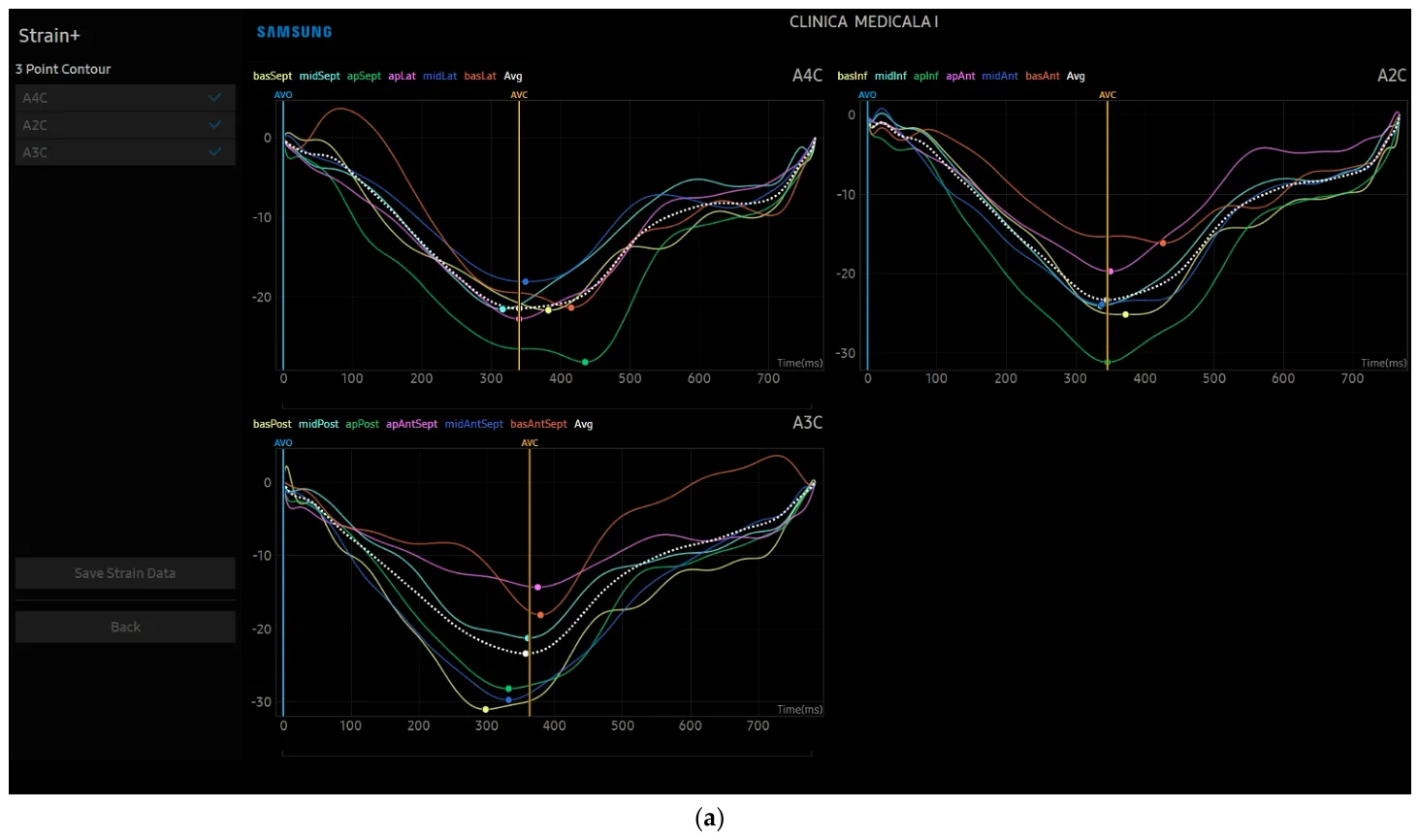

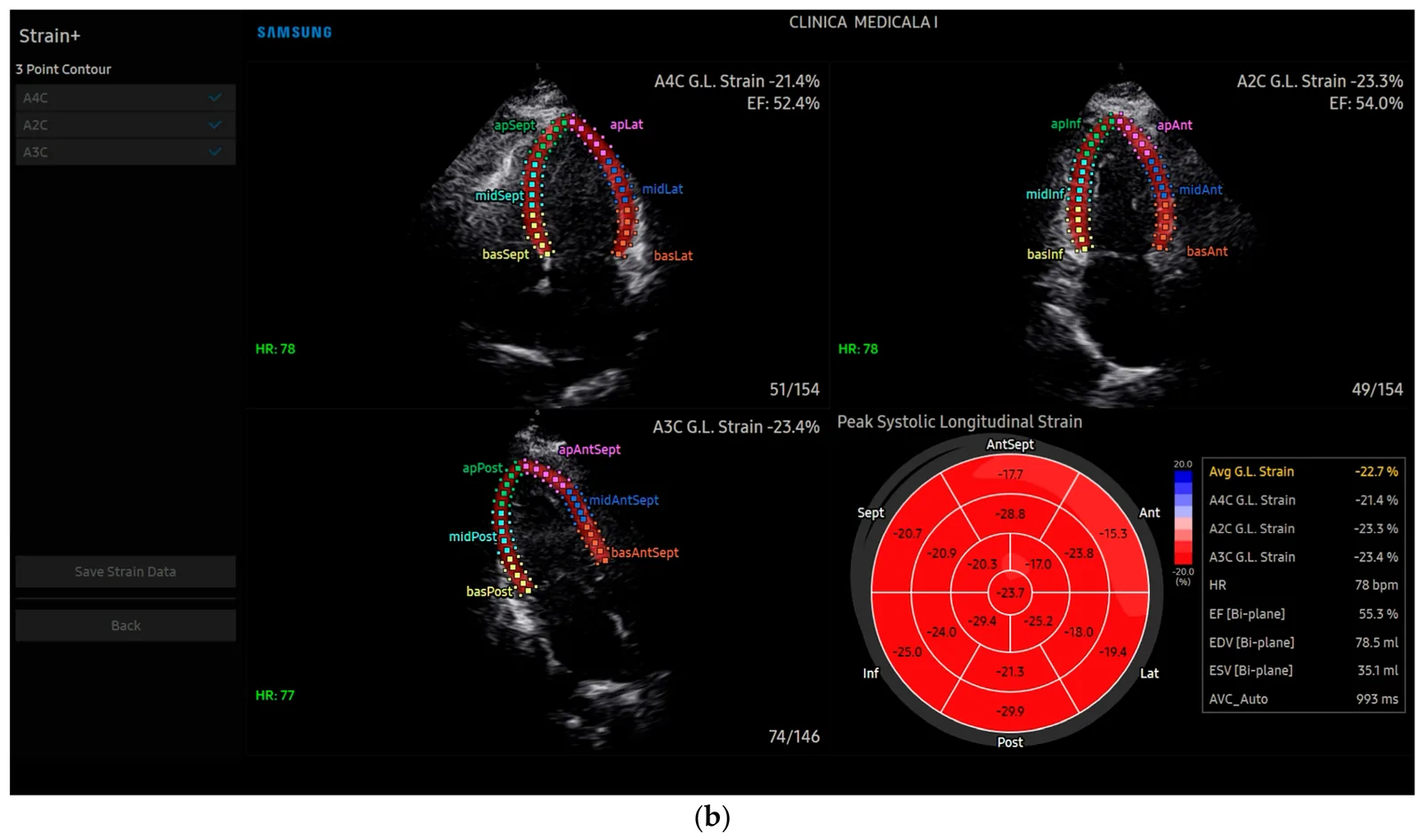
Left ventricular global longitudinal strain (Samsung cV8, Strain+), in a participant with stage 2 CKM syndrome. (**a**) Segmental strain-time curves from the apical four-, two-, and three-chamber views. (**b**) Endocardial tracking, 17-segment bull’s-eye. Global longitudinal strain −22.7%, biplane ejection fraction 55.3%.

**Figure 3:**
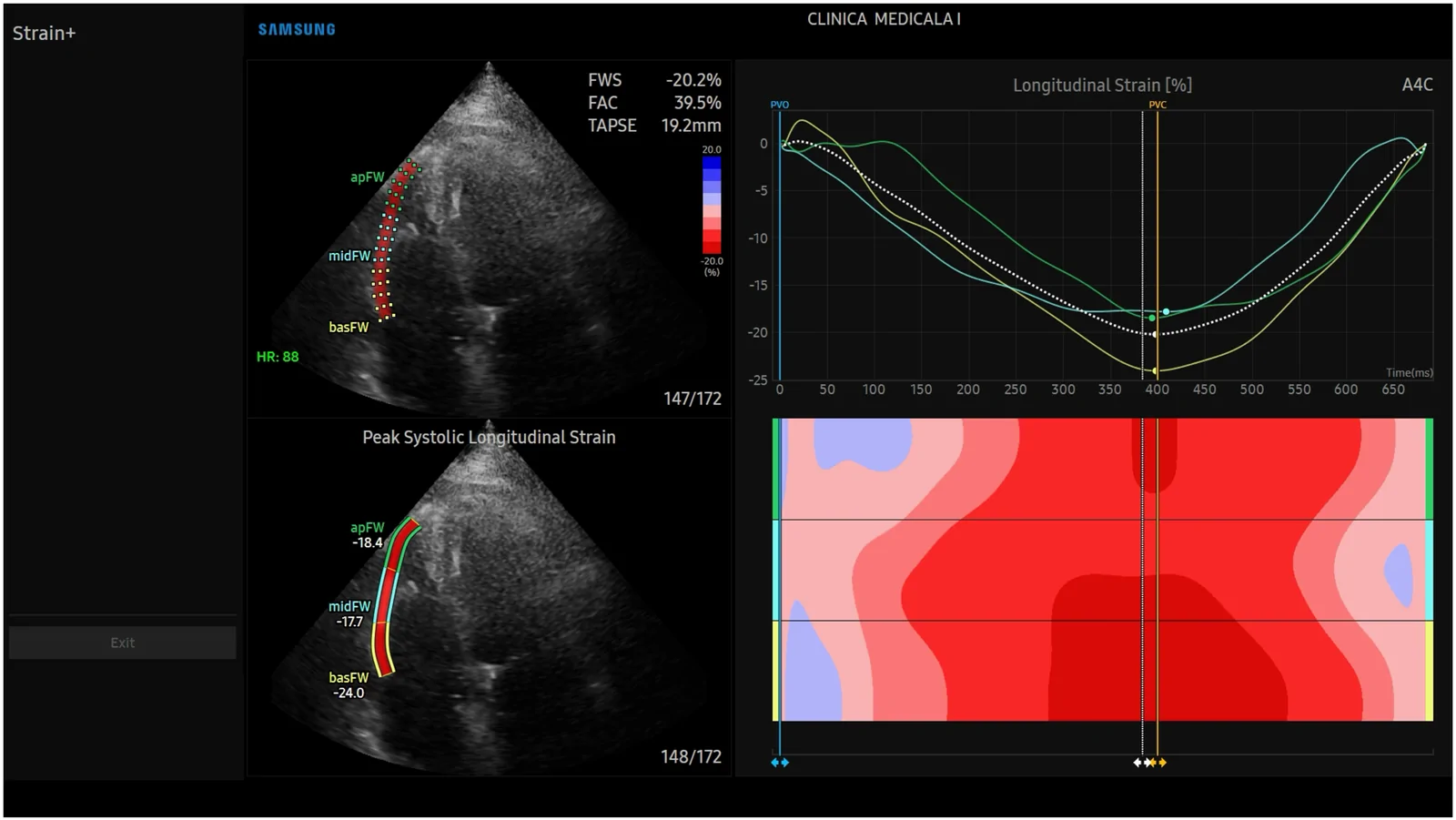
Right ventricular free-wall longitudinal strain (Samsung cV8, Strain+) from the right-ventricle-focused apical four-chamber view. Free-wall strain −20.2%, fractional area change 39.5%, tricuspid annular plane systolic excursion (TAPSE) 19.2 mm. Strain-time curves and segment labels: basal (basFW, yellow), mid (midFW, cyan), and apical (apFW, green) free-wall segments; dotted white curve, average free-wall strain. Vertical lines mark pulmonary valve opening (PVO, blue) and pulmonary valve closure (PVC, orange). Lower left: peak systolic longitudinal strain of each segment; lower right: color-coded segmental strain over time (red, shortening; blue, lengthening).

**Figure 4:**
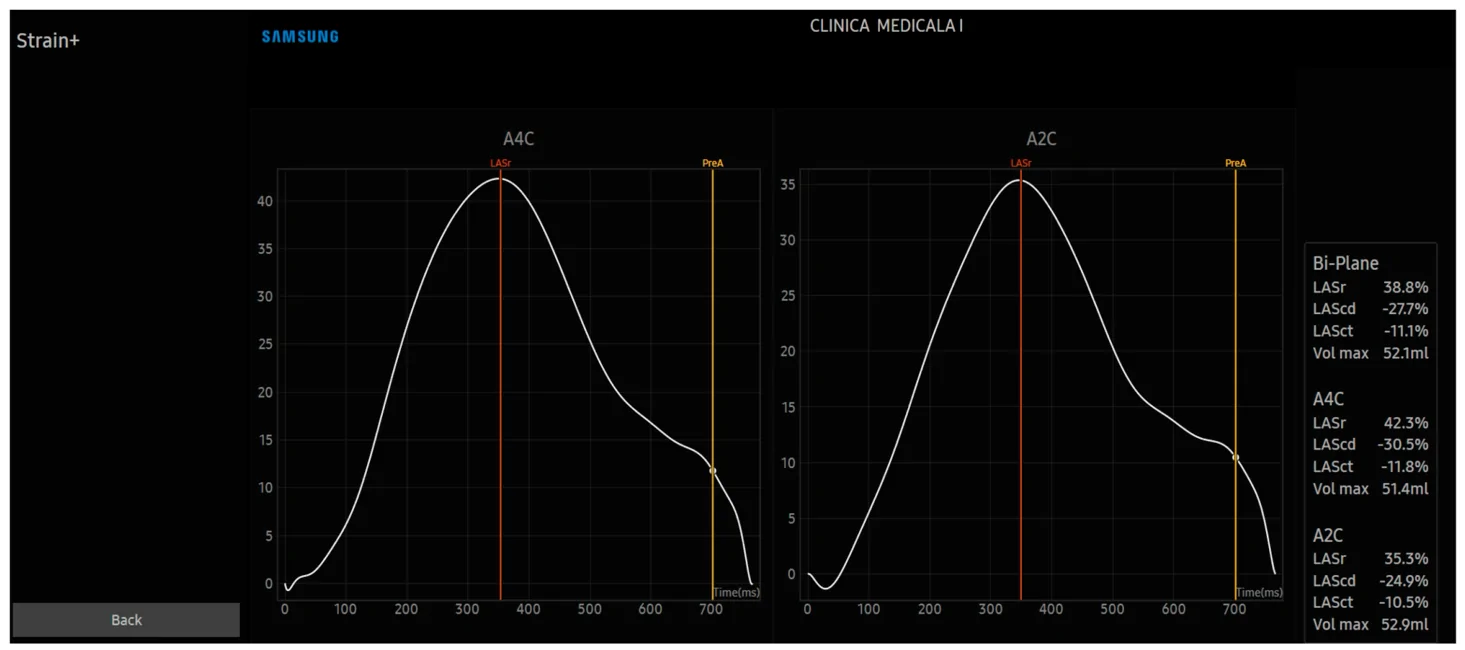
Left atrial strain (Samsung cV8, Strain+). Strain-time curves from apical four- and two-chamber views, with the reservoir (LASr) and pre-atrial-contraction (PreA) points marked. Components are reported as a biplane average: LASr 38.8%, LAScd −27.7%, and LASct −11.1%.

Left ventricular longitudinal strain is measured across the three apical views (Figure 2a,b). Right ventricular free-wall strain is measured from the right-ventricle-focused apical four-chamber view (Figure 3). Left atrial strain is measured with ventricular end-diastole as the reference, reporting LASr, LAScd, and LASct components for each view and as a biplane average (Figure 4). In participants who are in AF at the index visit, only the reservoir strain is reported, as the absence of atrial systole does not allow measurement of the conduit and contractile phases; participants in the established-AF cohort who are in sinus rhythm at the index visit have all three components measured.

Two-dimensional speckle-tracking echocardiography derives myocardial deformation from the acoustic markers–the “speckles” generated as ultrasound interacts with tissue [22]. The software follows these patterns frame by frame in the grayscale image and computes regional shortening and lengthening. It does not require an electrocardiogram; automated implementations can locate end-diastole and end-systole from the image alone. On the Samsung cV8, this is handled by Strain+, which places the endocardial border automatically, produces the strain-time curves, and displays the results for the left ventricle, the left atrium, and the right ventricular free wall. The operator inspects each tracing and corrects the border where needed before accepting it.

Rationale for LA strain. Reservoir strain measures the stretch of the atrial wall during ventricular systole and reflects its compliance and contractile reserve rather than chamber size; for this reason, it declines while the LAVI is still normal. In CKM syndrome, where inflammation, pressure load, and early fibrosis affect the atrium before it dilates, a reduced LASr identifies remodeling that volume-based criteria would classify as normal [23,24]. Reduced reservoir strain is associated with higher filling pressures and with subsequent AF, HFpEF, and stroke. As diastolic dysfunction progresses, the components change in a typical sequence: conduit strain decreases first, the contractile component compensates transiently, and the reservoir strain declines as the wall stiffens [25].

As part of routine acquisition quality control, any measurement considered uncertain at the time of analysis is remeasured by the same operator, and persistently inconclusive cases are reviewed by a second operator accredited by the European Association of Cardiovascular Imaging (EACVI). This ad hoc check performed during acquisition is distinct from the prespecified intra- and interobserver reproducibility analysis described in Section 4.9.

### 4.7. Cardiac Magnetic Resonance

In an exploratory sub-sample, atrial fibrosis is assessed by CMR. Scanning is done on a 3-tesla scanner (Siemens Healthineers, Erlangen, Germany), and LGE of the left atrium is analyzed with ADAS 3D software (ADAS3D Medical S.L., Barcelona, Spain). The software segments the atrial wall and estimates fibrosis from the image intensity ratio, which normalizes the wall signal to the blood pool so that values can be compared between patients [26]. Fibrosis is defined as an image intensity ratio above 1.2. Contraindications (implanted device, eGFR below 30 mL/min/1.73 m^2^, claustrophobia, refusal) and other reasons for not performing the examination are recorded; we expect to scan approximately 30 participants given the scanner capacity constraint. Scans are analyzed blinded to the electrocardiographic and echocardiographic data. Because LGE imaging is technically demanding in the thin atrial wall, it is quantified with ADAS 3D, which provides standardized three-dimensional atrial wall tissue characterization, thereby reducing the risk of partial-volume and segmentation effects. The availability of ADAS 3D for the whole study period cannot yet be guaranteed. If the software is not available, left atrial fibrosis will not be quantified, the fibrosis criterion will not be applied, and the CMR component may not be reported. ACM classification does not require CMR, because Step 2 can be met by the echocardiographic criteria, and no planned analysis depends on CMR (Section 3). The final report will state whether the fibrosis analysis was performed.

### 4.8. CKM Staging and ACM Classification

Once the clinical, laboratory, electrocardiographic, and imaging data are complete, each participant is assigned a CKM stage according to the AHA framework, and each participant in the sinus-rhythm cohort is classified as ACM-present, ACM-absent, or indeterminate according to Figure 1. Borderline electrocardiographic markers are adjudicated by a second reader.

### 4.9. Data Management, Quality, and Timeline

Data are entered on the electronic case report form with built-in range and consistency checks, and the main variables are verified against source documents. Quality-control reviews are carried out every two to three months, and the database is locked once these are complete. The project runs over 24 months, with month 1 corresponding to April 2026: setup and ethics approval (months 1–3), recruitment and baseline assessment (months 4–19; July 2026 to October 2027), ongoing quality control and database lock (months 5–20), analysis and reporting (months 20–22), and dissemination (months 22–24).

Reproducibility is quantified for the measurements that contribute directly to the ACM classification, that is, the principal electrocardiographic indices described in Section 4.3 and the echocardiographic parameters described in Section 4.5 and Section 4.6, in a subset of 20 participants of the sinus-rhythm cohort selected from the study database using computer-generated random numbers. At least four weeks after the original reading, the first operator repeats the measurements on the same stored electrocardiographic tracings and image loops, blinded to the original values, and a second operator, EACVI-accredited for the echocardiographic measurements, measures the same tracings and loops independently, blinded to the first operator’s results. The electrocardiographic measurements reassessed are P-wave duration, P-wave axis, P-wave amplitude, terminal force in lead V1, and the grading of Bayés interatrial block; the echocardiographic measurements are LAVI and the left atrial, left ventricular, and right ventricular strain components. Agreement for each continuous parameter is reported as an intraclass correlation coefficient with 95% confidence interval (absolute agreement, single measures; two-way mixed-effects model for intraobserver and two-way random-effects model for interobserver agreement) and as Bland–Altman limits of agreement, separately for the intraobserver and interobserver comparisons; agreement for the categorical grading of interatrial block is reported as Cohen’s kappa with 95% confidence interval.

### 4.10. Ethics

The study is conducted in accordance with the Declaration of Helsinki, Good Clinical Practice, and applicable local regulations. Ethics approval was obtained from the institutional review board before the start of the study, and the approval documentation is available on request. Participation is voluntary, and patients may withdraw at any time without any effect on their care. Personal and clinical data are pseudonymized, encrypted, and access-controlled in line with the General Data Protection Regulation. Non-invasive testing is prioritized throughout.

## 5. Expected Results

We expect the prevalence of ACM to be higher at more advanced CKM stages, and this stage-by-stage prevalence, with confidence intervals, will be the primary result. Comparing the two groups should identify the clinical, laboratory, ECG, and echocardiographic features associated with the atrial phenotype; we anticipate that reservoir strain will be lower at more advanced stages, including in participants whose LA volume index is still within the normal range. We will also relate Bayés interatrial block to CKM stages, examine the association of MR-proANP, Fetuin-A, FGF-23, and NT-proBNP with ACM and CKM category, and describe LA remodeling in users and non-users of SGLT2 inhibitors or GLP-1 receptor agonists. Because exposure is not randomized and duration of treatment is not recorded, this comparison is exploratory.

Significance, limitations, and future directions. The main strength of the design is also what makes it feasible: it uses only measurements already obtained in routine cardiology, with ACM definition and analysis plan fixed in advance. CMR is used as an advanced tissue-characterization tool for atrial fibrosis in an exploratory subset. The use of non-invasive tools could allow the identification of factors associated with ACM and support its earlier detection in a population frequently encountered in current practice [27]. Several limitations apply. The cross-sectional, single-visit design can identify associations but not sequence or causation, and cannot report outcomes such as incident stroke, AF, or heart failure, which are left to a later longitudinal phase. The single-center design limits generalizability and may introduce referral bias. Because participants with previously documented AF are allocated to the established-AF cohort irrespective of the rhythm at the index visit, the sinus-rhythm cohort excludes a group in whom ACM is likely to be advanced; the prevalence estimate therefore refers to patients without documented AF and is likely to be conservative with respect to the wider CKM population. Participants who are in AF at the index visit are less completely phenotyped, and the whole established-AF cohort is analyzed outside the primary analyses. Given the sample size, the analysis of individual parameters is exploratory and hypothesis-generating, providing a basis for further evaluation in larger, multicenter cohorts. As no universally accepted diagnostic standard for ACM exists, the criteria used here are among several reasonable choices, and participants are therefore also classified under alternative schemes. Deformation values are vendor-specific and will require confirmation on other systems, and CMR is available in only part of the cohort, with quantification of atrial fibrosis dependent on the availability of dedicated software. These limitations define the next steps: validation in a larger cohort, longitudinal follow-up to test whether non-invasively defined ACM predicts events, and extension to a national CKM registry.

## Data Availability

The data presented in this study are available on request from the corresponding author due to privacy and ethical restrictions, as the data are part of an ongoing study. The statistical analysis code (IBM SPSS Statistics 31.0 syntax) and the data dictionary will be made available on publication of the primary results.

## Supplementary Materials

The following supporting information can be downloaded at: https://www.mdpi.com/article/10.3390/diagnostics16193196/s1, Figure S1: Planned participant flow diagram for the ATRIO-CKM study; Table S1: Schedule of assessments for the ATRIO-CKM study; Table S2: STROBE-based reporting checklist for the ATRIO-CKM protocol.

## Abbreviations

The following abbreviations are used in this manuscript:

ACE	angiotensin-converting enzyme
ACM	atrial cardiomyopathy
AF	atrial fibrillation
AHA	American Heart Association
ARBs	angiotensin II receptor blockers
BMI	body mass index
CKM	cardiovascular-kidney-metabolic
CMR	cardiac magnetic resonance
EACVI	European Association of Cardiovascular Imaging
ECG	electrocardiogram
eGFR	estimated glomerular filtration rate
ELISA	enzyme-linked immunosorbent assay
ESC	European Society of Cardiology
FGF-23	fibroblast growth factor 23
GLP-1	glucagon-like peptide-1
HbA1c	glycated hemoglobin
HFA	Heart Failure Association
HFpEF	heart failure with preserved ejection fraction
LA	left atrium/left atrial
LAScd	left atrial conduit strain
LASct	left atrial contractile strain
LASr	left atrial reservoir strain
LAVI	left atrial volume index
LGE	late gadolinium enhancement
LV	left ventricle/left ventricular
MR-proANP	mid-regional pro-atrial natriuretic peptide
NT-proBNP	N-terminal pro-B-type natriuretic peptide
RV	right ventricle/right ventricular
SGLT2	sodium–glucose cotransporter 2
STROBE	Strengthening the Reporting of Observational Studies in Epidemiology
TAPSE	tricuspid annular plane systolic excursion
VIF	variance inflation factor
