# Supplementary material for: Atrial Cardiomyopathy in Patients with Cardiovascular-Kidney-Metabolic Syndrome: Non-Invasive Characterization (ATRIO-CKM)
Source: Diagnostics (Basel). 2026 Oct 1;16(19):3196. doi: 10.3390/diagnostics16193196 (PMC13664494; doi:10.3390/diagnostics16193196)
Supplement: Supplementary file 1 [file diagnostics-16-03196-s001.zip › Supplementary_TableS1_FigureS1.pdf]

Supplementary Table S1. Schedule of assessments for the ATRIO-CKM study.

All study assessments except cardiac magnetic resonance are performed at a single index visit, and no follow-up visits are planned. Cohort allocation follows the definition of established AF (Section 2.4) and not the rhythm recorded at the index visit: participants with AF documented at any time, including previously documented paroxysmal AF, are allocated to the established-AF cohort. Within that cohort, the extent of phenotyping depends on the rhythm at the index visit. Apart from cardiac magnetic resonance, which is scheduled as scanner capacity permits, the only procedure performed after the index visit is the offline reproducibility reassessment of stored electrocardiographic tracings and echocardiographic image loops (Section 4.9).

**Assessment**	**Timing**	**Sinus-rhythm cohort (no documented AF)**	**Established-AF cohort (AF documented at any time)**	**Protocol section**
Eligibility screening and screening log	Index visit	Yes	Yes	4.1
Written informed consent	Index visit, before any study-specific procedure	Yes	Yes	4.1
Demographics, clinical history, drug history	Index visit	Yes	Yes	4.2
Physical examination (BMI, abdominal perimeter, blood pressure, heart rate)	Index visit	Yes	Yes	4.2
CKM stage assignment	Index visit	Yes	Yes	2.4, 4.8
Resting 12-lead ECG (rhythm; AF pattern if present)	Index visit	Yes	Yes	4.3
P-wave duration, axis, amplitude, terminal force in V1, dispersion; Bayés interatrial block	Index visit	Yes	Only if in sinus rhythm at the index visit; not measurable if in AF	4.3, 2.8
Venous blood sampling: routine laboratory panel	Index visit	Yes	Yes	4.4
Study biomarkers (MR-proANP, Fetuin-A, FGF-23, NT-proBNP)	Index visit	Yes	Yes	4.4
Comprehensive transthoracic echocardiography	Index visit	Yes	Yes	4.5
LV global longitudinal strain; RV free-wall strain	Index visit	Yes	Yes	4.6
LA volume index	Index visit	Yes	Yes	4.5, 4.6

(LAVI); LA reservoir strain (LASr)				
LA conduit and contractile strain (LAScd, LASct)	Index visit	Yes	Only if in sinus rhythm at the index visit; not measurable if in AF	4.6, 2.8
ACM classification (Figure 1): ACM-present / ACM-absent / indeterminate	Index visit	Yes	Not applicable (analyzed as a separate cohort; those in sinus rhythm are described as a prespecified subgroup)	2.4, 2.7, 4.8
Cardiac magnetic resonance with late gadolinium enhancement (atrial fibrosis quantified only if the analysis software is available)	Index visit or as scheduling permits	Exploratory subset (~30 participants)	Exploratory subset	4.7
Intraobserver and interobserver reproducibility reassessment of the stored ECG tracings and echocardiographic loops	≥4 weeks after the original reading (offline)	Random subset of 20 participants	—	4.9

ACM, atrial cardiomyopathy; AF, atrial fibrillation; BMI, body mass index; CKM, cardiovascular-kidney-metabolic; ECG, electrocardiogram; FGF-23, fibroblast growth factor 23; LA, left atrial; LAScd, left atrial conduit strain; LASct, left atrial contractile strain; LASr, left atrial reservoir strain; LAVI, left atrial volume index; LV, left ventricular; MR-proANP, mid-regional pro-atrial natriuretic peptide; NT-proBNP, N-terminal pro-B-type natriuretic peptide; RV, right ventricular.

Supplementary Figure S1. Planned participant flow diagram for the ATRIO-CKM study.

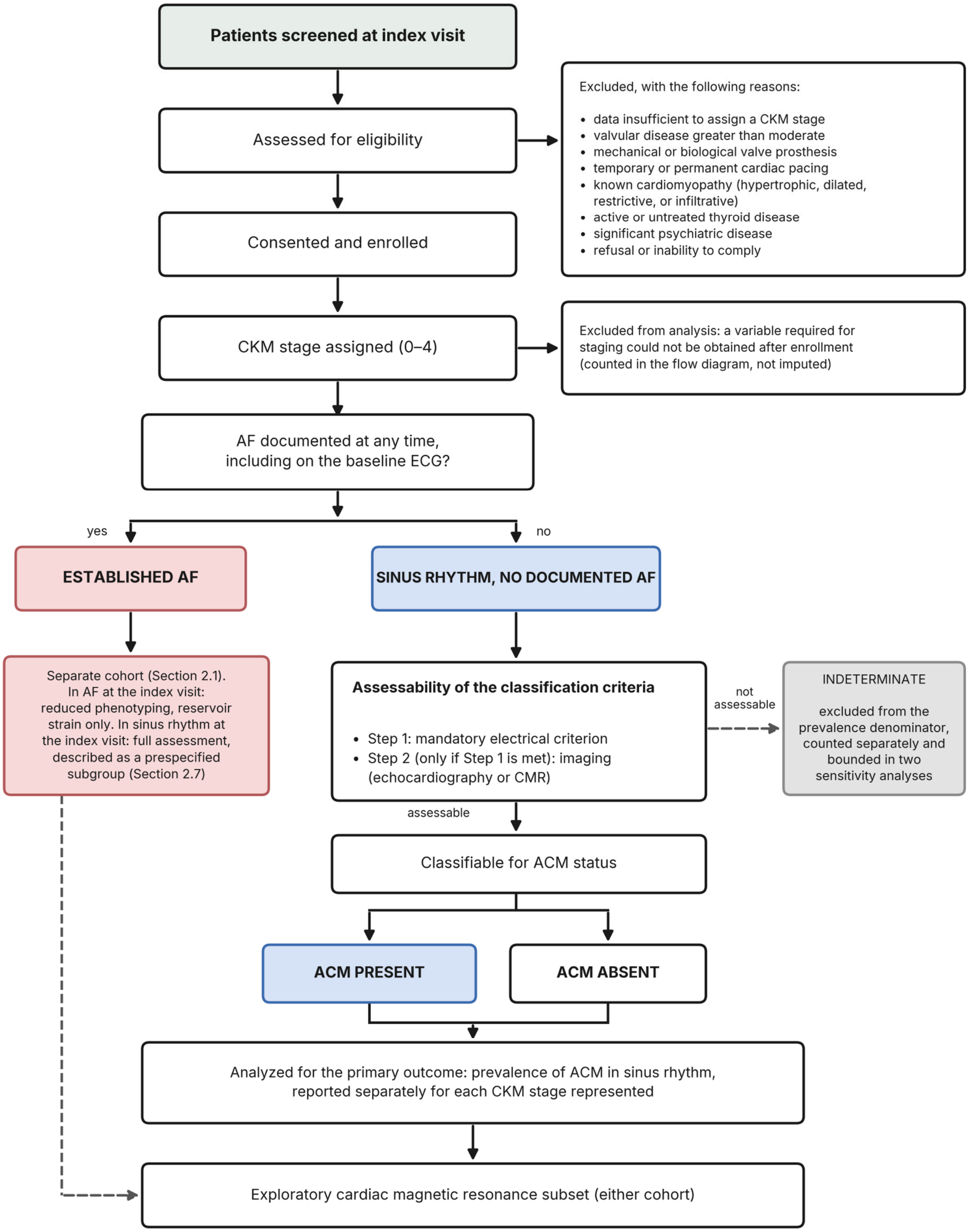

Planned structure of the participant flow diagram that will be reported with the primary results, showing screened patients, exclusions with reasons, cohort allocation by documented AF, the indeterminate category with its two prespecified sensitivity analyses, the ACM-present/ACM-absent classification, and the exploratory cardiac magnetic resonance subset. Participant counts will be added in the final study report. ACM, atrial cardiomyopathy; AF, atrial fibrillation; CKM, cardiovascular-kidney-metabolic; CMR, cardiac magnetic resonance; ECG, electrocardiogram.
