# Supplementary material for: Atrial Cardiomyopathy in Patients with Cardiovascular-Kidney-Metabolic Syndrome: Non-Invasive Characterization (ATRIO-CKM)
Source: Diagnostics (Basel). 2026 Oct 1;16(19):3196. doi: 10.3390/diagnostics16193196 (PMC13664494; doi:10.3390/diagnostics16193196)
Supplement: Supplementary file 1 [file diagnostics-16-03196-s001.zip › Supplementary_TableS2_STROBE_checklist.pdf]

Supplementary Table S2. STROBE-based reporting checklist for the ATRIO-CKM protocol

The STROBE statement is a reporting guideline for completed observational studies; no consensus reporting guideline for observational study protocols has yet been published in final form (the SPIROS checklist is still being finalized, and the SPIRIT statement applies to protocols of randomized trials). This checklist therefore maps the STROBE items to the present protocol, indicating for each item whether it is addressed here or is a results item that will be reported in the primary results paper in accordance with STROBE. Section numbers refer to the present version of the protocol.

Item	STROBE item	Addressed	Location in this protocol	Notes
1	Title and abstract	Yes	Title; Abstract	Design named in the title block ("Study Protocol") and in Abstract – Methods.
2	Background/rationale	Yes	1. Introduction	
3	Objectives	Yes	2.2. Objectives	
4	Study design	Yes	Abstract; 2.1. Study design and rationale	Single-center, prospective, observational, cross-sectional phenotyping.
5	Setting	Yes	2.1. Study design and rationale; 4.9. Data management, quality, and timeline	Registration NCT07697469; recruitment 10 July 2026 to October 2027; timeline in 4.9.
6	Participants	Yes	2.3. Population and eligibility	Eligibility criteria (2.3) and cohort allocation by documented AF, irrespective of the rhythm at the index visit (2.1, 2.4).
7	Variables	Yes	2.5. Outcome measures; 2.4. A priori definition of ACM and CKM staging	Outcomes, exposures and the a priori ACM/CKM definitions.
8	Data sources/ measurement	Yes	4.2–4.7 (Detailed Procedure); 3. Materials and Equipment	Per-modality methods; intraobserver and interobserver reproducibility of the principal P-wave and echocardiographic measurements prespecified in 4.9.
9	Bias	Yes	5. Expected Results; 2.1. Study design and rationale; 4.7. Cardiac magnetic resonance; 4.9. Data management, quality, and timeline	Referral bias and single-center generalizability discussed in 5; AF cohort analyzed separately (2.1); blinding of CMR reads (4.7) and of the intra-/interobserver electrocardiographic and echocardiographic reassessment (4.9) as concrete design-stage bias-

				mitigation measures.
10	Study size	Yes	2.6. Sample size	
11	Quantitative variables	Yes	2.7. Statistical analysis; 2.4. A priori definition of ACM and CKM staging	Handling of continuous variables and the prespecified cut-offs.
12	Statistical methods	Yes	2.7. Statistical analysis; 2.8. Missing data	Includes the prespecified model assumption and diagnostic checks, the handling of multiple comparisons, the missing-data strategy with the variables eligible for imputation, and the complete-case and indeterminate-status sensitivity analyses.
13	Participants (flow)	Planned; structure prespecified	Figure S1 (cited in 2.4); Supplementary Materials; 2.8. Missing data	Flow structure defined in advance in Figure S1; counts to be reported in the results paper.
14	Descriptive data	Planned	2.7. Statistical analysis	
15	Outcome data	Planned	2.5. Outcome measures; 2.7. Statistical analysis	
16	Main results	Planned	2.7. Statistical analysis	Effect measures and confounder handling prespecified.
17	Other analyses	Planned	2.7. Statistical analysis; 2.8. Missing data	Subgroup and sensitivity analyses, including imputed versus complete-case.
18	Key results	Planned	5. Expected Results	
19	Limitations	Yes	5. Expected Results; 2.4; 2.8	Vendor-specific strain offsets, indeterminate cases, missing-at-random assumption, availability of CMR fibrosis quantification (4.7).
20	Interpretation	Yes	5. Expected Results	
21	Generalisability	Yes	5. Expected Results	
22	Funding	Yes	Funding	Internal Research Grants competition 2025 (contract no. 7386/2026).
